# Synthesis of the tetracyclic core of *Illicium* sesquiterpenes using an organocatalyzed asymmetric Robinson annulation

**DOI:** 10.3762/bjoc.9.126

**Published:** 2013-06-12

**Authors:** Lynnie Trzoss, Jing Xu, Michelle H Lacoske, Emmanuel A Theodorakis

**Affiliations:** 1 Department of Chemistry and Biochemistry, University of California, San Diego, 9500 Gilman Drive, La Jolla, CA 92093-0358, USA

**Keywords:** natural products, neurodegenerative diseases, neurotrophic small molecule, organocatalysis, total synthesis

## Abstract

An enantioselective synthesis of the core framework of neurotrophic *Illicium* majucin-type sesquiterpenes is described here. This strategy is based on an organocatalyzed asymmetric Robinson annulation and provides an efficient approach for a diversity-oriented synthesis of *Illicium* natural products that holds remarkable therapeutic potential for neurodegenerative diseases.

## Introduction

Neurotrophins are a family of endogenous proteins that are vital for neuron function, survival, and regeneration [1–3]. As such, they have prompted intense studies toward the treatment of various neurodegenerative diseases including Alzheimer’s disease [4] and Parkinson’s disease [5]. Despite their unambiguous importance, approaches to neurotrophin-based drug development have encountered problems associated with their limited oral availability, insufficient delivery to the central neural system and considerable manufacturing cost [6–7]. These limitations have stimulated the search for small molecules that can enhance or mimic neurotrophin activity as potential drug leads [8–12].

**Figure 1:**
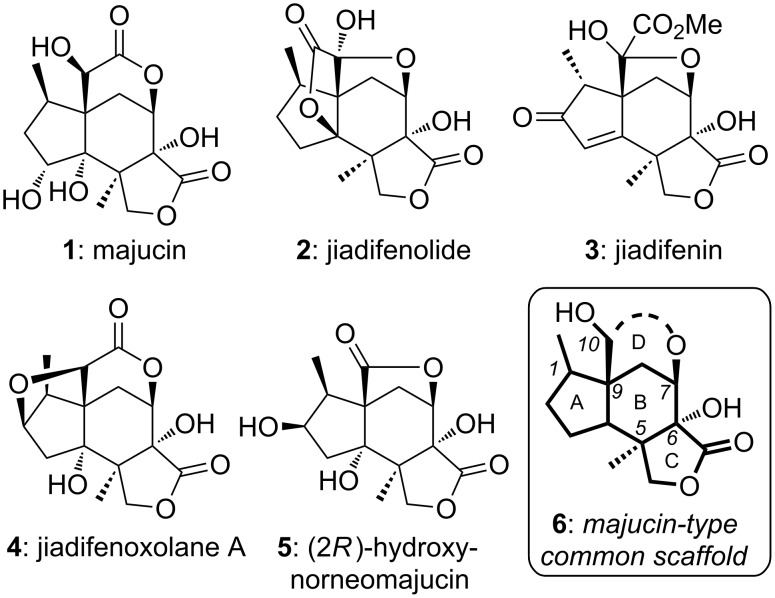
Representative majucin-type *Illicium* sesquiterpenes.

Majucin-type *Illicium* sesquiterpenes (Figure 1) [13], such as majucin (**1**) [14–15], jiadifenolide (**2**) [16], jiadifenin (**3**) [17], jiadifenoxolane A (**4**) [16] and (2*R*)-hydroxynorneomajucin (**5**) [18], share a caged tetracyclic scaffold (**6**). These compounds (**2**–**5**) have shown a great potential in enhancing neurite outgrowth in primary cultured rat cortical neurons at low nanomolar to low micromolar concentrations. Thus, to develop an efficient synthetic approach toward the complex core skeleton of these natural products is of paramount importance. Consequently, this family of neurotrophic sesquiterpenes has been the focus of extensive synthetic studies in which asymmetric and efficient construction of the tetracyclic core presents the principal challenge [19–23].

**Figure 2:**
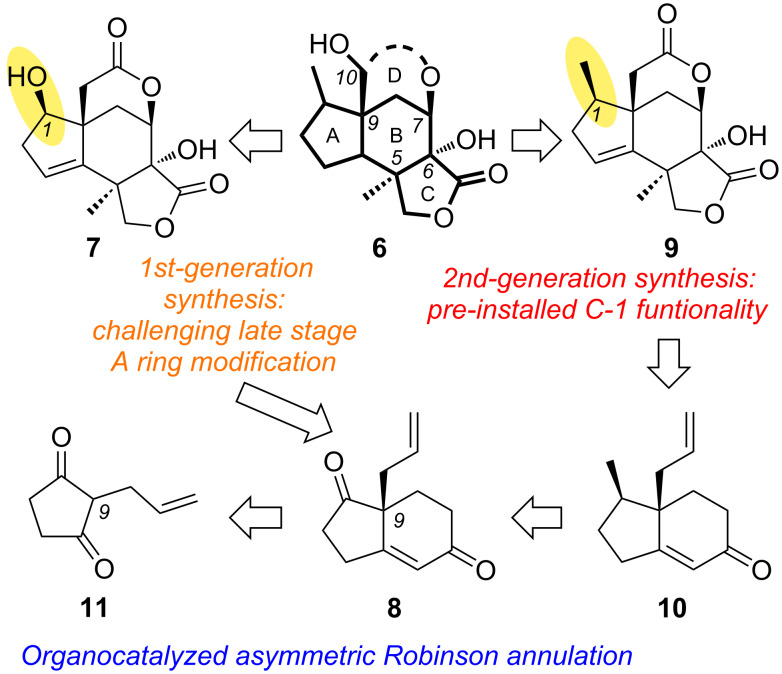
Comparison of core skeleton synthetic strategies.

We have recently reported a unified synthetic strategy of **2**, **3** and designed analogues using scaffold **7** as the key intermediate (Figure 2) [24–26]. A potential drawback of this strategy is the late-stage modification of the A ring motif of **7** that requires additional steps for the synthesis of the target molecules. In an effort to overcome this issue, we describe here a second-generation strategy of framework **9** in which the C-1 center has been methylated early in the synthesis. As such, it represents an efficient route toward a diversity-oriented synthesis of several *Illicium* sesquiterpenes. The enantioselective entry to these molecules is based on an organocatalyzed asymmetric Robinson annulation that allows access to the enantiomerically enriched bicyclic motif **8** from achiral diketone **11** (Figure 2).

## Results and Discussion

**Scheme 1:**
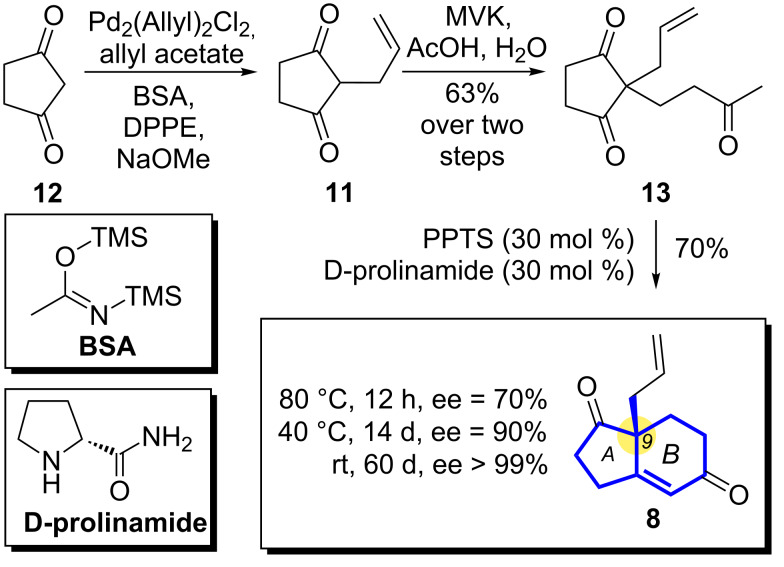
Organocatalyzed asymmetric Robinson annulation.

During the past 20 years, organocatalysis has emerged as an important field in asymmetric stereoselective synthesis due to its advantages, which include high enantioselectivity, environmental friendliness and ease of handling [27–50]. Organocatalyzed asymmetric Robinson annulation has long been proven to be one of the most powerful strategies to construct bicyclic systems with a chiral quaternary center [51–58]. Among them, the Hajos–Wiechert and Wieland–Miescher ketones represent two of the most famous examples [59–65]. With this background information in mind, we devised an enantioselective synthesis of **8** starting from commercially available dione **12**, and the synthesis of **8** was previously published [25–26]. Tsuji–Trost allylation [66–68] of **12** produced compound **11**, which was readily converted to **13** by an acid-catalyzed Michael addition with methyl vinyl ketone (MVK) (two steps, 63% overall yield) [69–71]. The organocatalyzed cyclization of **13** was achieved by optimizing the previously reported Tu/Zhang conditions [71] using D-prolinamide as the organocatalyst (Scheme 1). Performing this reaction at 80 °C gave rise to bicyclic motif **8** in about 70% ee (70 % yield after 12 h), while decreasing the temperature to 25 °C increased the enantioselectivity to over 99% (70% yield after 60 days). To compromise between high enantioselectivity and short reaction time, we decided to pursue this conversion at 40 °C where we obtained an enantiomeric excess of 90% (70% yield after 14 days).

**Scheme 2:**
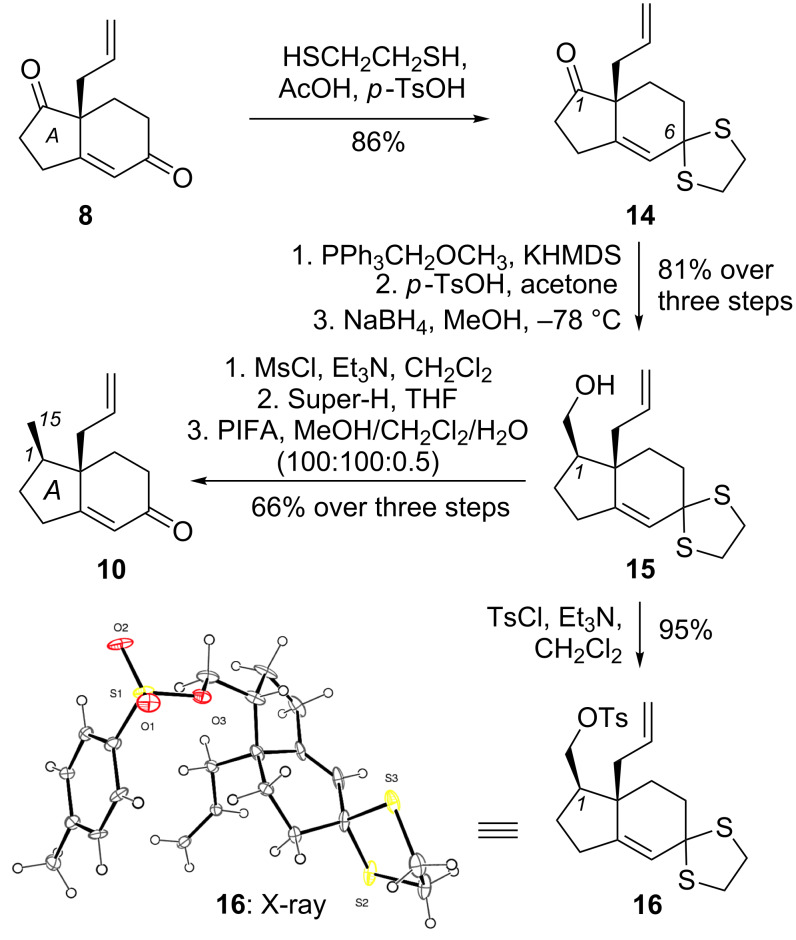
Early stage A-ring functionalization.

The enantiomerically enriched Hajos–Wiechert-like diketone **8** (ee > 90%) was then subjected to a selective protection of the C-6 enone motif to yield dithioketal **14** (86% yield) [72–74]. Wittig olefination of the C-1 ketone with methoxymethylenetriphenylphosphine [75] yielded the corresponding enol methyl ether, which was hydrolyzed to the aldehyde under acidic conditions and reduced with NaBH_4_ to form alcohol **15** with desired diastereoselectivity at the C-1 center (dr = 9:1) in 81% yield (over three steps) [76]. The stereochemistry of **15** was unambiguously confirmed by single-crystal X-ray analysis of the related tosylate derivative **16** [77]. Deoxygenation of the C-15 primary alcohol was performed by: (a) mesylation of the alcohol with MsCl; and (b) reductive deoxygenation with LiEt_3_BH (super hydride). The thioketal protecting group was then removed under oxidative conditions with [bis(trifluoroacetoxy)iodo]benzene (PIFA) to yield ketone **10** in good yield (66% over three steps, Scheme 2) [78]. This approach allowed us to produce a sufficient amount of enone **10** (>10 grams) for further functionalization.

**Scheme 3:**
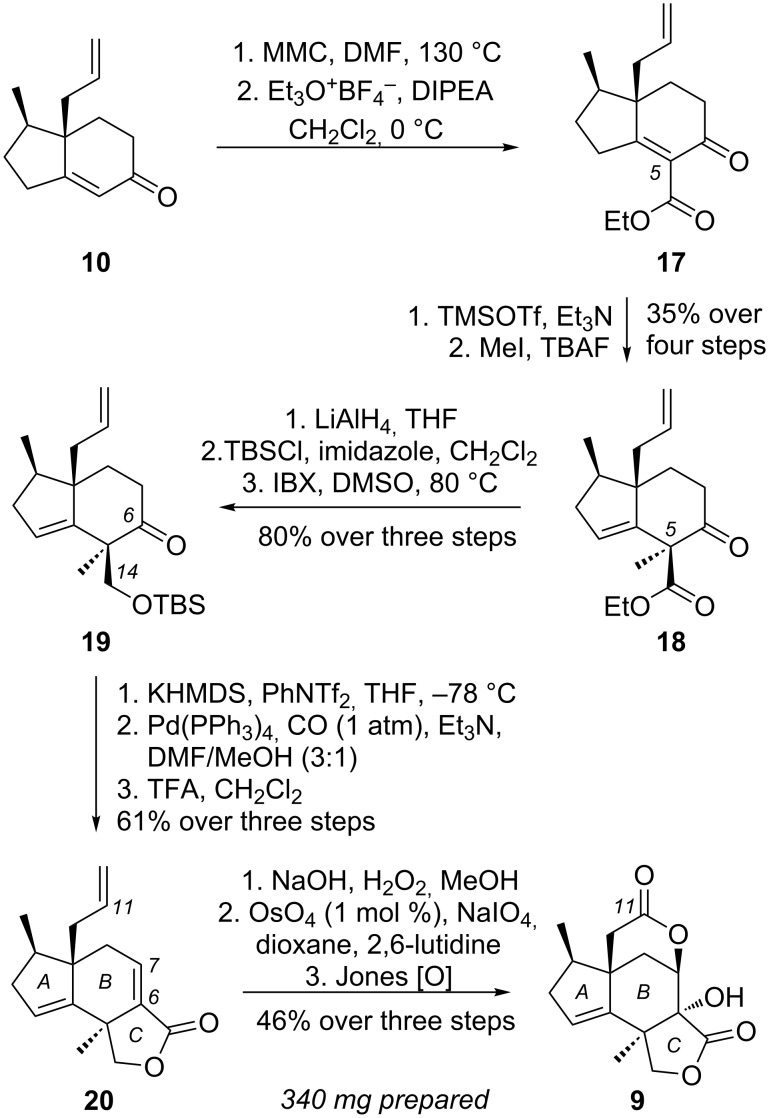
Synthesis of core scaffold **9**.

Conversion of **10** to **9** was accomplished based on our previously reported strategy (Scheme 3) [25]. Treatment of **10** with magnesium methyl carbonate (MMC) [79–81] yielded the C-5 carboxylic acid that, without further purification, was esterified under Meerwein’s conditions to afford β-ketoester **17**. Treatment of **17** with TMSOTf/Et_3_N followed by enolate alkylation [82] under TBAF/MeI conditions afforded the desired C-5 quaternary center of **18** as a single isomer (35% over four steps). Global reduction of **18** with lithium aluminium hydride produced the corresponding C-6/C-14 diol motif. Selective TBS protection of the C-14 primary alcohol followed by an IBX oxidation of the C-6 secondary alcohol yielded ketone **19** in 80% combined yield over three steps. Triflation of the C-6 ketone with McMurry’s reagent (PhNTf_2_) [83–86] followed by a Pd(0)-catalyzed carbomethoxylation [87–90] produced the desired C-ring lactone **20** in 61% yield. Epoxidation of the C-6/C-7 enone with NaOH/H_2_O_2_ followed by oxidative cleavage of the C-11 terminal alkene under OsO_4_/NaIO_4_ conditions [91–92] afforded the corresponding C-11 aldehyde. Exposure of this intermediate to Jones oxidation triggered a highly efficient oxidation–epoxide opening [93–98] reaction cascade [99–100] to construct the critical D-ring of **9** (46% yield, over 3 steps). Notably, this scalable approach rendered us several hundred milligrams of compound **9**, paving the way for a diversity-oriented synthesis. For example, a Mn(III) promoted C-2 allylic oxidation [24,101–102] would provide a C-2 oxygenated functionality. Similarly, C-10 α-substitution would provide a large diversity of neurotrophic analogues based on our recent findings [26].

## Conclusion

We describe here an efficient and enantioselective approach to tetracyclic lactone **9** representing a key motif toward the synthesis of various neurotrophic [103–110] *Illicium* sesquiterpenes. Key to the strategy was a highly enantioselective Robinson annulation reaction that proceeded under organocatalytic conditions to form the Hajos–Wiechert-like enone **8**. The overall strategy highlights the importance of organocatalytic approaches in the modern synthesis of bioactive natural products [111–116].

## Supporting Information

File 1 Experimental procedures for the syntheses of all new compounds.
