# Supplementary material for: Synthesis of the tetracyclic core of Illicium sesquiterpenes using an organocatalyzed asymmetric Robinson annulation
Source: Beilstein J Org Chem. 2013 Jun 12;9:1135–40. doi: 10.3762/bjoc.9.126 (PMC3701413; doi:10.3762/bjoc.9.126)
Supplement: File 1 — Experimental procedures for the syntheses of all new compounds. [file Beilstein_J_Org_Chem-09-1135-s001.pdf]

Supporting Information

for

Synthesis of the tetracyclic core of *Illicium* sesquiterpenes using an organocatalyzed asymmetric Robinson annulation

Lynnie Trzoss, Jing Xu, Michelle H. Lacoske and Emmanuel A. Theodorakis*

Address: Department of Chemistry and Biochemistry, University of California, San Diego, 9500 Gilman Drive, La Jolla, CA 92093-0358, USA

Email: Emmanuel A. Theodorakis - etheodor@ucsd.edu

* Corresponding author

Experimental procedures for the syntheses of all new compounds

General procedures.

Unless indicated, all commercially available reagents and anhydrous solvents were purchased at the highest commercial quality and were used as received without further purification. All nonaqueous reactions were carried out under an argon atmosphere using dry glassware that had been flame-dried under a stream of argon unless otherwise noted. Dry tetrahydrofuran (THF), diethyl ether (Et_2O), methylene chloride (CH_2Cl_2) and dimethylformamide (DMF) were obtained by passing commercially available predried, oxygen-free formulations through activated alumina columns. Flash column chromatography was performed on silica gel (Merck Kieselgel 60, 230–400 mesh) using hexane/EtOAc or CH_2Cl_2/MeOH mixtures of increasing polarity. The progress of all the reactions was monitored by thin-layer chromatography (TLC) using glass plates precoated with silica gel-60 F254 to a thickness of 0.5 mm (Merck). ^{13}C NMR and ^{1}H NMR spectra were recorded on either 400 MHz/500 MHz Varian instrument or a 500 MHz JEOL instrument. Chemical shifts (δ) are quoted in parts per million (ppm) referenced to the appropriate residual solvent peak, with the abbreviations s, br s, d, t, q, and m denoting singlet, broad singlet, doublet, triplet, quartet and multiplet respectively. *J* = coupling constants given in hertz (Hz). High-resolution mass spectra (HRMS) were recorded on a trisector WG AutoSpecQ spectrometer. Optical rotation data were collected on a Jasco P-1010 polarimeter using HPLC grade anhydrous solvents. X-ray data were recorded on a Bruker SMART APEX 3kW Sealed Tube X-ray diffraction system.

14: To a vigorous stirred solution of **8** [1,2] (10.0 g, 52.6 mmol) in glacial AcOH (20 mL), was added at room temperature a solution of ethane-1,2-dithiol (5.45 g, 57.8 mmol) and *p*-TsOH (1.71 g, 15.8 mmol) in glacial AcOH (40 mL). The mixture was stirred for 2 h and was quenched by water (100 mL). The solid was separated by filtration and washed with water (50 mL), with 10% aqueous solution of $NaHCO_3$ solution (50 mL), and with water (50 mL), and dried under reduced pressure. The crude product was then dissolved in EtOAc, rotavaped on silica and purified by a plug of silica (hexane/EtOAc = 100:5) to afford **14** as white crystals (12 g, 86%); $[\alpha]_D^{25}$ –373.9 (*c* 0.6, $CHCl_3$); ^{1}H NMR (400 MHz, $CDCl_3$) δ 5.76 (s, 1H), 5.71 (m, 1H), 5.11 (m, 2H), 3.42 (m, 3H), 3.23 (m, 1H), 2.69 (m, 1H), 2.52 (m, 2H), 2.24 (m, 5H), 1.96 (m, 1H), 1.50 (m, 1H); ^{13}C NMR (100 MHz, $CDCl_3$) δ 217.4, 142.3, 132.8, 126.5, 118.8, 64.9, 51.1, 40.9, 39.9, 38.5, 37.8, 36.5, 26.6; HRMS (ESI): m/e 267.0871 [M + H^+] calcd for $C_{14}H_{19}OS_2^+$: 267.0872.

15: To a suspension of $Ph_3P^+CH_2OCH_3Cl^-$ (27.8 g, 81 mmol) in anhydrous THF (400 mL) at 0 °C, was added a solution of KHMDS (90 mL, 90 mmol, 1 M in THF). The resulting red solution was stirred at the same temperature for 30 minutes, and a solution of ketone **14** (12.0 g, 45 mmol) in THF (100 mL) was added slowly. The mixture was then warmed up to rt and left overnight. Upon completion, the reaction was quenched by saturated NH_4Cl solution (200 mL) and brine (200 mL). The residue was extracted with EtOAc (3 × 200 mL) and dried over Na_2SO_4. The solvent was removed under reduced pressure, and the crude product was purified through a plug of silica (hexane/EtOAc = 10:1 as elute) to afford the corresponding enol ether as a yellow oil. The methyl enol

ether obtained above (~13.5 g, 45 mmol) was dissolved in acetone (1.5 L). To this solution was added *p*-TsOH (25.6 g, 134 mmol) at 0 °C. The reaction was then warmed up to rt and stirred for 2 h. Upon completion, the reaction was quenched with saturated $NaHCO_3$ solution (30 mL) and the solvent was removed by rotovap (water bath temperature below 30 °C). The residue was diluted with water (400 mL) and extracted with EtOAc (3 × 200 mL). The combined organic layers were washed with brine (200 mL) and dried over Na_2SO_4. The solvent was then removed and the crude aldehyde was isolated as a 9:1 mixture of diastereomers at C1, which was used without further purification. Aldehyde obtained as described above (~12.5 g, 45 mmol) was dissolved in MeOH (300 mL) and THF (300 mL) at −78 °C, and $NaBH_4$ was added in small portions. The resulting suspension was stirred at the same temperature for 30 minutes before being quenched with saturated NH_4Cl solution (100 mL). The solvent was removed by rotovap (water bath temperature below 30 °C). The residue was diluted with water (300 mL) and extracted with EtOAc (3 × 200 mL). The combined organic layers were washed with brine (100 mL) and dried over Na_2SO_4. The solvent was removed and the crude alcohol was purified by flash column chromatography (silica, hexane/EtOAc = 4:1) to afford **15** as a yellow oil (10.2 g, 81% over 3 steps); $[\alpha]_D^{25}$ –60.7 (*c* 1.6, $CHCl_3$); ^1H NMR (500 MHz, $CDCl_3$) δ 5.78 (m, 1H), 5.52 (s, 1H), 5.08 (d, *J* = 18.9 Hz, 1H), 5.00 (d, *J* = 5.9 Hz, 1H), 3.76 (dd, *J* = 10.4 Hz, 7.5 Hz, 1H), 3.69 (dd, *J* = 10.9 Hz, 7.5 Hz, 1H), 3.38 (m, 3H), 3.22 (m, 1H), 2.43–2.28 (m, 2H), 2.26 (m, 1H), 2.15–2.11 (m, 4H), 1.79 (m, 1H), 1.72 (m, 1H), 1.53 (m, 1H), 1.43 (m,1H), 1.23 (m, 1H); ^{13}C NMR (100 MHz, $CDCl_3$) δ 149.0, 136.5, 123.3, 116.9, 65.9, 63.9, 54.2, 44.9, 40.5, 39.8, 38.9, 37.0, 34.8, 28.2, 24.7; HRMS (ESI): *m/e* 283.1186 [M + H^+] calcd for $C_{15}H_{23}OS_2^+$: 283.1185.

16: To a solution of alcohol **15** (20 mg, 71 µmol) in anhydrous DCM (350 µL) was added Et_3N (20 µL, 142 µmol) and TsCl (25 µg, 135 µmol) at 0 °C. The reaction was then warmed up to rt and left overnight. Upon completion, brine (3 mL) and pH 7 buffer solution (3 mL) was added, and the residue was extracted with CH_2Cl_2 (3 × 5 mL). The combined organic extracts were dried over Na_2SO_4, and the solvent was removed under reduced pressure. The crude product was then purified by flash column chromatography (silica, hexane/EtOAc = 10:1) to afford tosylate **16** as white crystal. (30 mg, 95%); $[\alpha]_D^{25}$ – 68.9 (*c* 1.0, $CHCl_3$); ^{1}H NMR (500 MHz, $CDCl_3$) δ 7.79 (d, *J* = 8.1 Hz, 2H), 7.36 (d, *J* = 8.1 Hz, 2H), 5.64 (m, 1H), 5.52 (s, 1H), 5.08 (d, *J* = 7.5 Hz, 1H), 4.93 (bs, 1H), 4.11 (dd, *J* = 9.7 Hz, 6.0 Hz, 1H), 4.09 (dd, *J* = 9.2 Hz, 6.9 Hz, 1H), 3.39 (m, 3H), 3.21 (m, 1H), 2.45 (s, 3H), 2.38–2.13 (m, 3H), 2.10 (m, 1H), 2.02 (m, 3H), 1.85 (m, 2H), 1.50 (dt, *J* = 13.8 Hz, 2.9 Hz, 1H), 1.31 (m, 1H); ^{13}C NMR (125 MHz, $CDCl_3$) δ 147.2, 144.9, 135.7, 133.0, 129.9, 128.0, 124.2, 117.0, 70.8, 65.4, 50.2, 44.8, 40.4, 39.7, 38.6, 36.8, 34.6, 27.9, 24.3, 21.7; HRMS (ESI): *m*/*e* 437.1275 [M + H^+] calcd for $C_{22}H_{29}O_3S_3^+$: 437.1273.

10: To a solution of alcohol **15** (10.2 g, 36.1 mmol) in anhydrous CH_2Cl_2 (180 mL) was added Et_3N (10.2 mL, 72.2 mmol) and MsCl (5.35 mL, 68.6 mmol) at 0 °C. The reaction was then warmed up to rt and stirred for an additional 30 minutes. Upon completion, brine (80 mL) and pH 7 buffer solution (80 mL) was added, and the residue was extracted with CH_2Cl_2 (3 × 50 mL). The combined organic extracts were dried over Na_2SO_4 and the solvent was removed under reduced pressure. The crude product was used for the

next step without future purification. Mesylate obtained above (~13 g, 36.1 mmol) was dissolved in anhydrous THF (150 mL). Super-Hydride® (181 mL, 1 M in THF, 181 mmol) was added slowly at 0 °C via cannula. The reaction was then warmed up to rt and left to stir for 24 h before being quenched by saturated NH_4Cl solution (50 mL) and diluted by brine (200 mL). The organic layer was separated and the aqueous layer was extracted with EtOAc (3 × 100 mL). The combined organic extracts were dried over Na_2SO_4 and concentrated. The crude product obtained as described above (~10 g, 36.1 mmol) was dissolved in MeOH (150 mL), DCM (150 mL) and water (0.75 mL). PIFA (23.2 g, 54.0 mmol) was then added in small portions. Upon complete addition, the reaction was stirred at rt for an additional 15 minutes before Na_2SO_3 (4.5 g, 36 mmol) was added. The reaction was stirred for 15 minutes and quenched by water (100 mL) and the excess solvent was removed under reduced pressure. The residue was extracted with CH_2Cl_2 (3 × 100 mL) and the combined organic extracts were washed with brine and dried over Na_2SO_4. The solvent was then removed under reduced pressure and the crude product was purified by flash column chromatography (silica, hexane/EtOAc = 10:1) to afford enone **10** as 9:1 diastereomeric mixture at C-1 (4.5 g, 66% 3 steps). $[\alpha]_D^{25}$ –174.9 (*c* 1.0, $CHCl_3$); ^1H NMR (500 MHz, $CDCl_3$) δ 5.88 (s, 1H), 5.82 (m, 1H), 5.07 (d, *J* = 18.4 Hz, 1H), 5.02 (d, *J* = 10.4 Hz, 1H), 2.63 (m, 2H), 2.32 (m, 1H), 2.29 (m, 2H), 2.18 (m, 2H), 1.90 (m, 1H), 1.72 (m, 2H), 1.59 (m, 1H), 1.05 (d, *J* = 6.9 Hz, 3H), 0.84* (d, *J* = 7.5 Hz, 0.2H, diastereomer at C15); ^{13}C NMR (125 MHz, $CDCl_3$) δ 201.6, 179.8, 135.4, 122.4, 117.6, 47.6, 46.7, 36.4, 33.7, 33.5, 29.9, 29.2, 13.5; HRMS (ESI): *m*/*e* 191.1432 $[M + H^+]$ calcd for $C_{13}H_{19}O^+$: 191.1430.

18: To a solution of **10** (3.0 g, 15.8 mmol) in anhydrous DMF (35 mL) was added magnesium methyl carbonate (27.6 mL, 55.2 mmol, 2.0 M in DMF). This solution was degassed for 5 min under argon, then immersed in an oil bath which was preheated to 130 °C and stirred for 3 h. The reaction was cooled to 0 °C and poured in to a mixture of ice/2 N HCl (50 mL). Then this mixture was acidified to pH = 2~3 with 2 N HCl. Ether (200 mL) was added to form a two-phase clear solution. The aqueous phase was separated and re-extracted with ether (2 × 100 mL). The combined organic extracts were dried over Na_2SO_4 and concentrated under reduced pressure at 30 °C. The residue was dried on high-vacuum pump for 1 h to remove the trace of DMF to afford yellow oil. The crude product was dissolved in dry CH_2Cl_2 (35 mL), triethyloxonium tetrafluoroborate (23.6 mL, 23.6 mmol, 1 M in CH_2Cl_2) was added at 0 °C, and then DIPEA (5.5 mL, 31.5 mmol) was added dropwise. After 1 minute, TLC showed the completion of this reaction. Then this reaction was quenched with saturated NH_4Cl solution (30 mL), extracted with DCM (3 × 30 mL), washed with saturated $NaHCO_3$ solution and brine, dried over Na_2SO_4, and concentrated under reduced pressure. The unstable crude product **17** was used in the next step directly. To a solution of this crude ester (4.2 g, ~15.8 mmol) in dry DCM (60 mL) was added 2,6-lutidine (5.5 mL, 41.3 mmol) at 0 °C, followed by the addition of TMSOTf (5.7 mL, 13.6 mmol) dropwise. After 30 min, the reaction was diluted with hexanes (100 mL), quenched with 5% (not saturated!) $NaHCO_3$ solution (50 mL), extracted with hexanes (3 × 80 mL), the combined organic extracts were dried over Na_2SO_4 and concentrated under reduced pressure. The residue was further dried on high-vacuum pump for 10 min (no longer!). The unstable crude TMS-enol ether was dissolved in dry THF (60 mL), cooled to −78 °C, and methyl iodide (9.8 mL, 0.16 mol)

was added in, followed by the addition of TBAF solution dropwise (15.8 mL, 15.8 mmol, 1 M in THF). This reaction was then allowed to warm to rt slowly over 30 min, and stirred at rt for an extra 2 h before it was quenched with saturated NH_4Cl solution (50 mL). The mixture was extracted with EtOAc (3 × 80 mL), and the combined organic phase was washed with brine, dried over Na_2SO_4 and concentrated under reduced pressure. The residue was purified by silica flash column chromatography (hexanes/EtOAc = 100:1 to 10:1) to afford **18** as a yellow oil (1.5 g, 35% over 2 steps). $[\alpha]_D^{23}$ –257.0 (*c* 0.7, $CHCl_3$); ^{1}H NMR (500 MHz, $CDCl_3$) δ 5.86 (dd, *J* = 3.5 Hz, 1.7 Hz, 1H), 5.78 (m, 1H), 5.08 (d, *J* = 2.3 Hz, 1H), 5.00 (td, *J* = 9.7 Hz, 2.3 Hz, 1H), 4.15 (m, 1H), 4.08 (m, 1H), 2.80 (m, 1H), 2.40–2.36 (m, 2H), 2.28 (m, 1H), 2.17–2.02 (m, 2H), 1.98 (m, 2H), 1.51 (m, 1H), 1.48 (s, 3H), 1.21 (t, *J* = 7.5 Hz, 3H), 1.08 (d, *J* = 7.5 Hz, 3H); ^{13}C NMR (125 MHz, $CDCl_3$) δ 208.3, 172.3, 147.2, 136.4, 129.2, 116.5, 61.7, 58.8, 49.8, 47.6, 39.4, 36.8, 36.5, 35.7, 20.1, 13.9, 13.1; HRMS (ESI) *m*/*e* 299.1617 [M + Na^+] calcd for $C_{17}H_{24}O_3Na^+$: 299.1618.

19: To a solution of **18** (1.5 g, 5.4 mmol) in dry THF (30 mL) was added $LiAlH_4$ solution (21.7 mL, 43.4 mmol, 2M in THF) at 0 °C. This reaction was stirred for 30 min before it was carefully quenched with water (1.5 mL) followed by 15% NaOH solution (1.5 mL) and water (4.5 mL). The reaction mixture was allowed to warm up to room temperature and stirred for an additional 15 minutes before anhydrous $MgSO_4$ was added and the mixture diluted with ether (30 mL). The mixture was stirred for 15 minutes before filtration through Celite® and washed thoroughly with ether (200 mL). The solvent was then removed under reduce pressure and the crude diol was used in the next step directly.

The crude diol was dissolved in DCM (30 mL) and was cooled to 0 °C. Imidazole (0.74 g, 10.8 mmol) was added followed by the addition of TBSCl (0.90 g, 6.0 mmol). After 30 min, this reaction was quenched with saturated NH_4Cl solution (200 mL), extracted with DCM (3 × 50 mL), washed with brine and dried over Na_2SO_4, then concentrated under reduced pressure. The crude mono-TBS-ether was used in the next step directly. The crude mono-TBS-ether was dissolved in dry DMSO (30 mL), IBX (4.6 g, 16.3 mmol) was added, and this reaction was heated to 80 °C for 1 h. Upon completion, the reaction was cooled to rt, water (50 mL) was added, the reaction was filtered through Celite®, and the filtrates were extracted with EtOAc (3 × 50 mL). The combined organic extracts were then washed with brine, dried over Na_2SO_4, and concentrated under reduced pressure. The obtained residue was purified by silica flash column chromatography (hexanes/EtOAc = 100:1 to 20:1) to afford **19** as a yellow oil (1.52 g, 80% over 3 steps). $[\alpha]_D^{23}$ –29.3 (*c* 0.8, $CHCl_3$); ^{1}H NMR (500 MHz, $CDCl_3$) δ 5.84 (m, 1H), 5.66 (m, 1H), 5.07 (d, *J* = 16.6 Hz, 1H), 4.96 (d, *J* = 10.3 Hz, 1H), 3.68 (d, *J* = 4.0 Hz, 2H), 2.49 (m, 2H), 2.32 (m, 2H), 2.23 (m, 2H), 2.04 (m, 2H), , (1.58 m, 1H), 1.19 (s, 3H), 1.09 (d, *J* = 6.9 Hz, 3H), 0.85 (s, 9H), 0.007 (s, 3H), −0.0045 (s, 3H); ^{13}C NMR (125 MHz, $CDCl_3$) δ 214.0, 150.9, 136.9, 127.1, 116.2, 69.3, 54.9, 49.4, 48.0, 39.3, 37.1, 36.1, 34.4, 26.1, 21.1, 21.5, 13.3, −5.5; HRMS (ESI) *m*/*e* 349.2556 [M + H^+] calcd for $C_{21}H_{37}O_2Si^+$: 349.2557

20: To a solution of ketone **19** (1.52 g, 4.36 mmol) in dry THF (40 mL) was added $PhNTf_2$ (4.67 g, 13.1 mmol) at room temperature. KHMDS (21.8 mL, 21.8 mmol, 1 M in THF) was added dropwise at –78 °C and stirred for 30 min before being warmed up to rt

over 30 min. The reaction was quenched by solution with saturated NH_4Cl solution (10 mL) and extracted with EtOAc (3 × 30 mL). The combined organic phase was washed with brine, dried over Na_2SO_4, concentrated under reduced pressure, and purified by silica flash column chromatography (hexanes/EtOAc = 100:1) to afford the vinyl triflate as a white solid. (1.7 g, 81%). The vinyl triflate obtained above (1.7 g, 3.54 mmol) was dissolved in DMF/MeOH (27mL/9 mL, 3:1), $Pd(PPh_3)_4$ (41 mg, 35 μmol) and triethylamine (1.5 mL, 10.6 mmol) was added. This orange solution was degassed under an argon atmosphere for 5 min, followed by bubbling in carbon monoxide for 5 min. This solution was then heated to 70 °C for 1 h under a carbon monoxide atmosphere before it was concentrated under reduced pressure. The residue was passed through a short silica pad (hexanes/EtOAc = 50:1, 1000 mL), concentrated and redissolved in dry CH_2Cl_2 (35 mL), TFA (0.83 mL, 10.8 mmol) was added, and this reaction was stirred at rt for 5 h before it was quenched with saturated $NaHCO_3$ solution (15 mL). The mixture was extracted with CH_2Cl_2 (3 × 30 mL), the combined organic phase was washed with brine, dried over Na_2SO_4, concentrated under reduced pressure and purified by silica flash column chromatography (hexanes/EtOAc = 100:1 to 10:1) to afford the lactone **20** as a white solid (0.65 g, 61% from **19**). $[\alpha]_D^{23}$ –28.1 (*c* 0.3, CHCl3); ^{1}H NMR (500 MHz, $CDCl_3$) δ 6.89 (dd, *J* = 3.1 Hz, 2.9 Hz, 1H), 5.77 (m, 1H), 5.66 (m, 1H), 4.96 (m, 2H), 4.14 (d, *J* = 8.0 Hz, 1H), 3.94 (d, *J* = 8.6 Hz, 1H), 2.52 (dd, *J* = 16.1 Hz, 7.5 Hz, 1H), 2.49 (m, 1H), 2.10 (m, 4H), 2.05 (dd, *J* = 16.0 Hz, 2.9 Hz, 1H), 1.33 (s, 3H), 1.12 (d, *J* = 6.9 Hz, 3H); ^{13}C NMR (125 MHz, $CDCl_3$) δ 169.8, 149.5, 135.9, 135.1, 134.8, 127.3, 117.0, 76.3, 53.9, 46.9, 41.7, 39.8, 37.0, 36.5, 27.7, 13.7; HRMS (ESI) *m*/*e* 267.1358 [M + Na^+] calcd for $C_{16}H_{20}O_2Na^+$: 267.1356.

9: To a solution of lactone **20** (0.65 g, 2.66 mmol) in MeOH (20 mL) was added a premixed solution of 3 N NaOH (2.5 mL) and 30% H_2O_2 (2.5 mL) dropwise at 0 °C. This reaction was warmed up to rt and vigorous stirred for 5 h. The mixture was then diluted with water, acidified with 2 N HCl to pH = 1, and separated with EtOAc/brine (20 mL/20 mL), and the aqueous phase was extracted with EtOAc (2 × 20 mL). The organic phase was combined, dried over Na_2SO_4, and concentrate under reduced pressure to afford epoxide as a white solid. The product was used without future purification. Epoxide obtained above (~0.7 g, 2.66 mmol) was dissolved in 1,4-dioxane (30 mL) and water (10 mL). To this solution 2,6-lutidine (0.63 mL, 5.4 mmol) and OsO_4 (0.17 mL, 4% solution in H_2O, 27 µmol) were added, and then $NaIO_4$ (2.3 g, 10.8 mmol) was added portionwise at 0 °C. This reaction was then warmed up to rt and stirred overnight. The reaction was diluted with water (60 mL) and extracted with CH_2Cl_2 (3 × 50 mL). The organic phase was dried over Na_2SO_4 and concentrated under reduced pressure to afford the aldehyde as a white solid, which was clean enough to be used for the next reaction. To a solution of the aldehyde obtained above (~0.7 g, 2.66 mmol) in acetone (20 mL) was added Jones reagent (2.3 mL, 6.2 mmol, 2.67 M) dropwise at 0 °C, and this reaction was stirred at 0 °C for 30 min. Ethanol (10 mL) was carefully added dropwise to quench this reaction, followed by dropwise addition of the saturated $NaHCO_3$ solution (10 mL). The mixture was stirred for 5 min before filtration through Celite®, and the filter cake was then washed thoroughly with EtOAc (100 mL). The combined organic extracts were dried over Na_2SO_4 and concentrated under reduced pressure. The crude residue was purified by column chromatography (hexanes/CH_2Cl_2 = 1:1 to 1:3 to 100% CH_2Cl_2, then

CH_2Cl_2/MeOH = 200:1 to 50:1) to afford the product **9** as a white foam (340 mg, 46% over 3 steps) $[\alpha]_D^{22}$ –20.7 (*c* 0.3, $CHCl_3$); ^{1}H NMR (500 MHz, CD_3OD) δ 5.95 (appeared as t, *J* = 5.6 Hz, 1H), 4.71 (dd, *J* = 2.9 Hz, 1.7 Hz, 1H), 3.99 (d, *J* = 9.8 Hz, 1H), 3.73 (d, *J* = 10.3 Hz, 1H), 2.69 (d, *J* = 18.3 Hz, 1H), 2.40 (m, 2H), 2.16 (dd, *J* = 13.8 Hz, 4.6 Hz, 1H), 2.06 (m, 2H), 1.85 (m, 1H), 1.30 (s, 3H), 1.04 (d, J = 6.9 Hz, 3H); ^{13}C NMR (125 MHz, $CDCl_3$) δ 177.5, 171.3, 146.5, 129.3, 129.1, 79.9, 76.7, 75.3, 44.9, 44.6, 42.0, 37.1, 29.5, 21.2, 12.4; HRMS (ESI) *m*/*e* 301.1048 [M + Na^+] calcd for $C_{15}H_{18}O_5Na^+$: 301.1046.

References:

[1] Xu, J.; Trzoss, L.; Chang, W.K.; Theodroakis, E. A. *Angew. Chem. Int. Ed.* **2011**, *50*, 3672-3676.

[2] Trzoss, L.; Xu, J.; Lacoske, M. H.; Mobley, W. C.; Theodorakis, E. A. *Chem. Eur. J.* **2013**, *20*, 6398-6408.

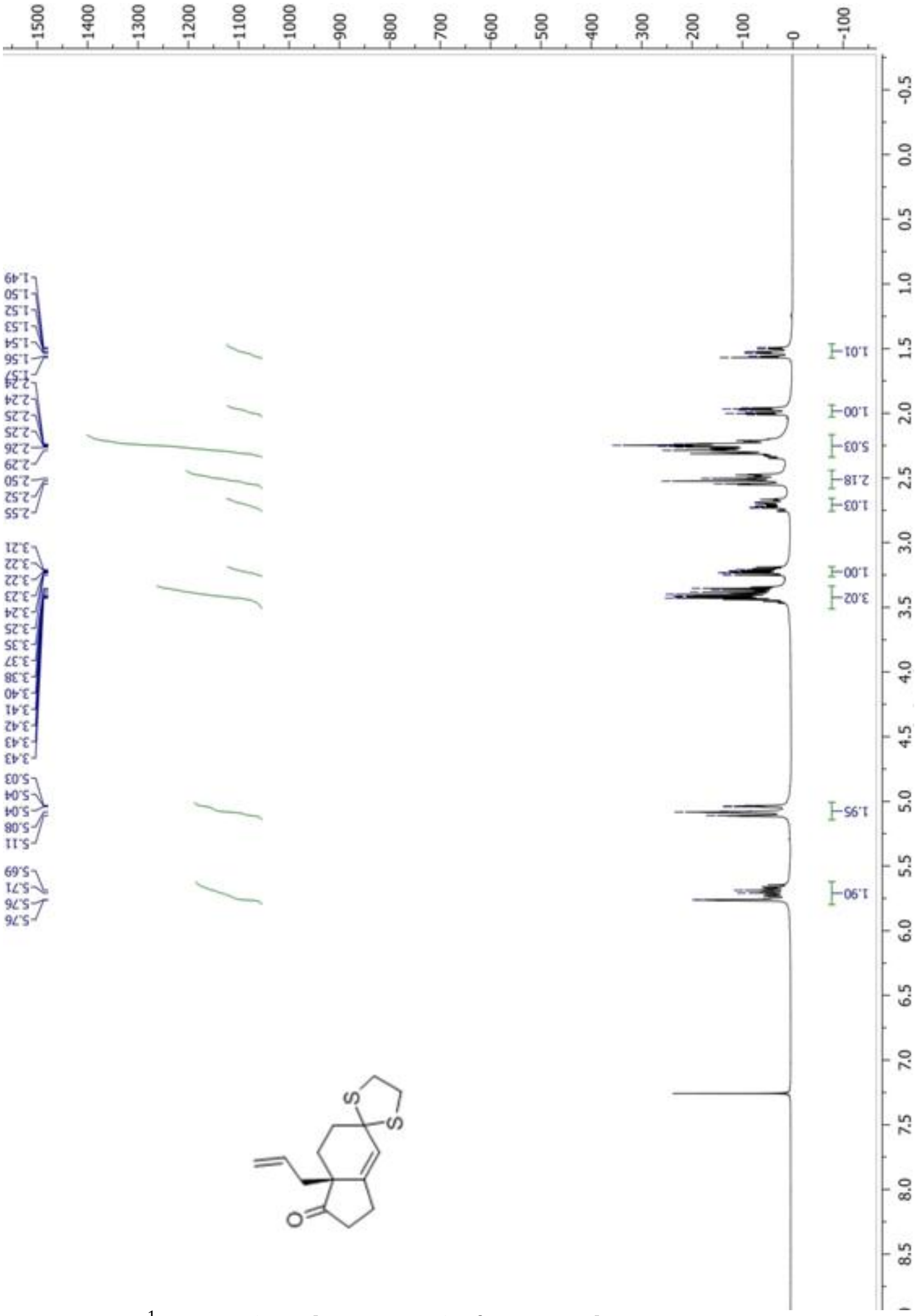

Spectrum 1. ^{1}H NMR ($CDCl_3$, 400 MHz) of compound **14**.

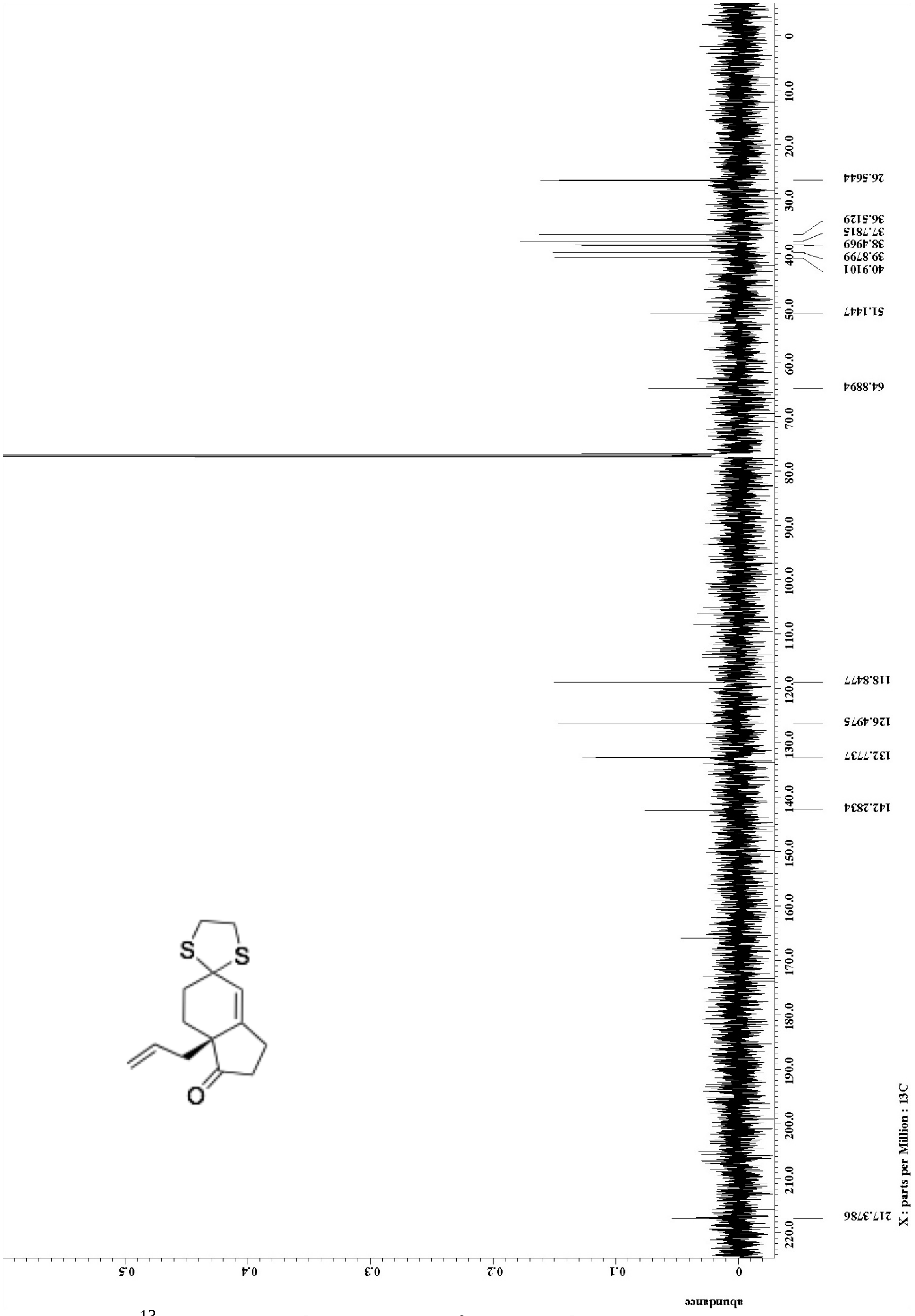

Spectrum 2. ^{13}C NMR ($CDCl_3$, 400 MHz) of compound **14**.

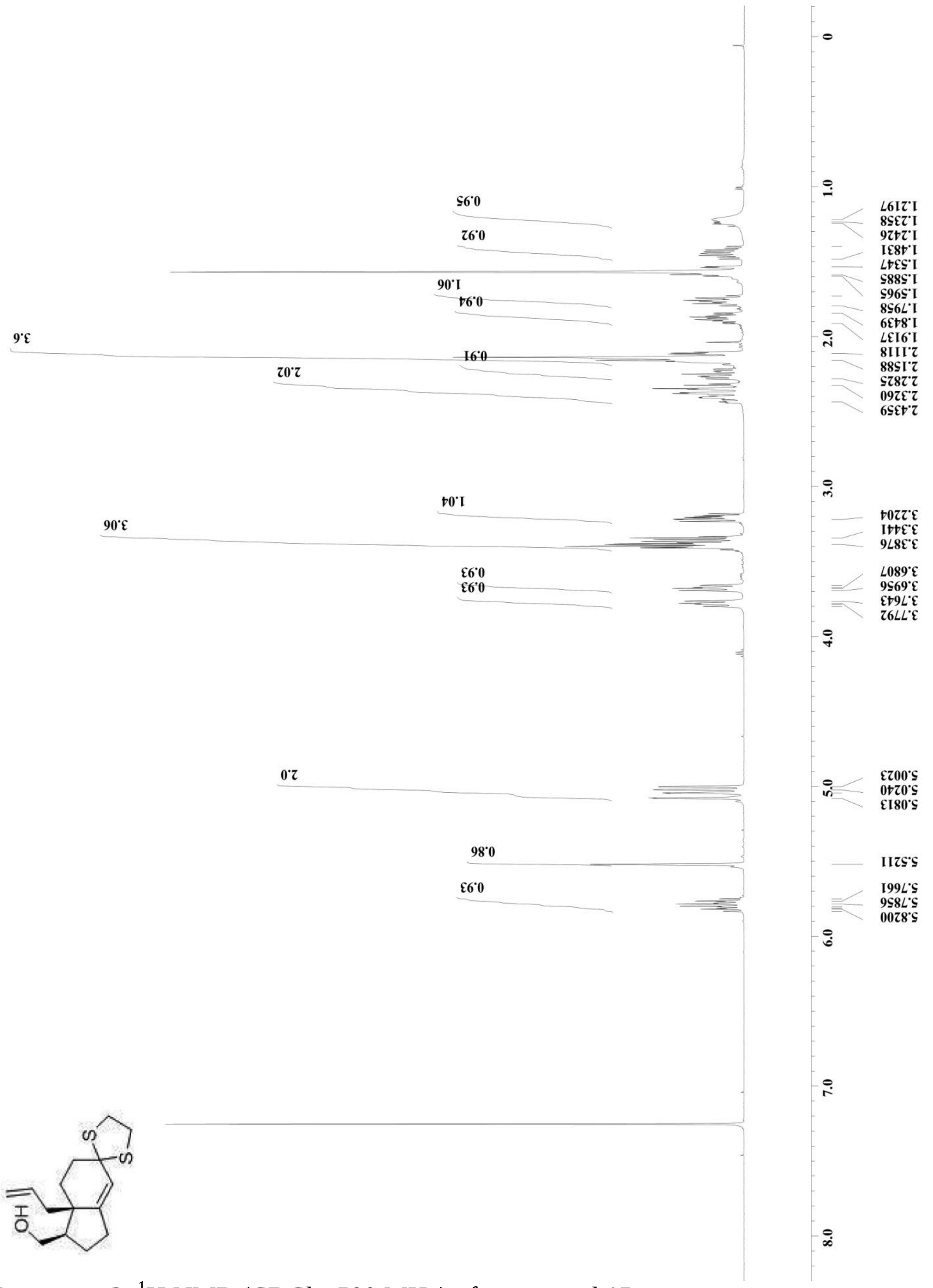

Spectrum 3. ^{1}H NMR ($CDCl_3$, 500 MHz) of compound **15**.

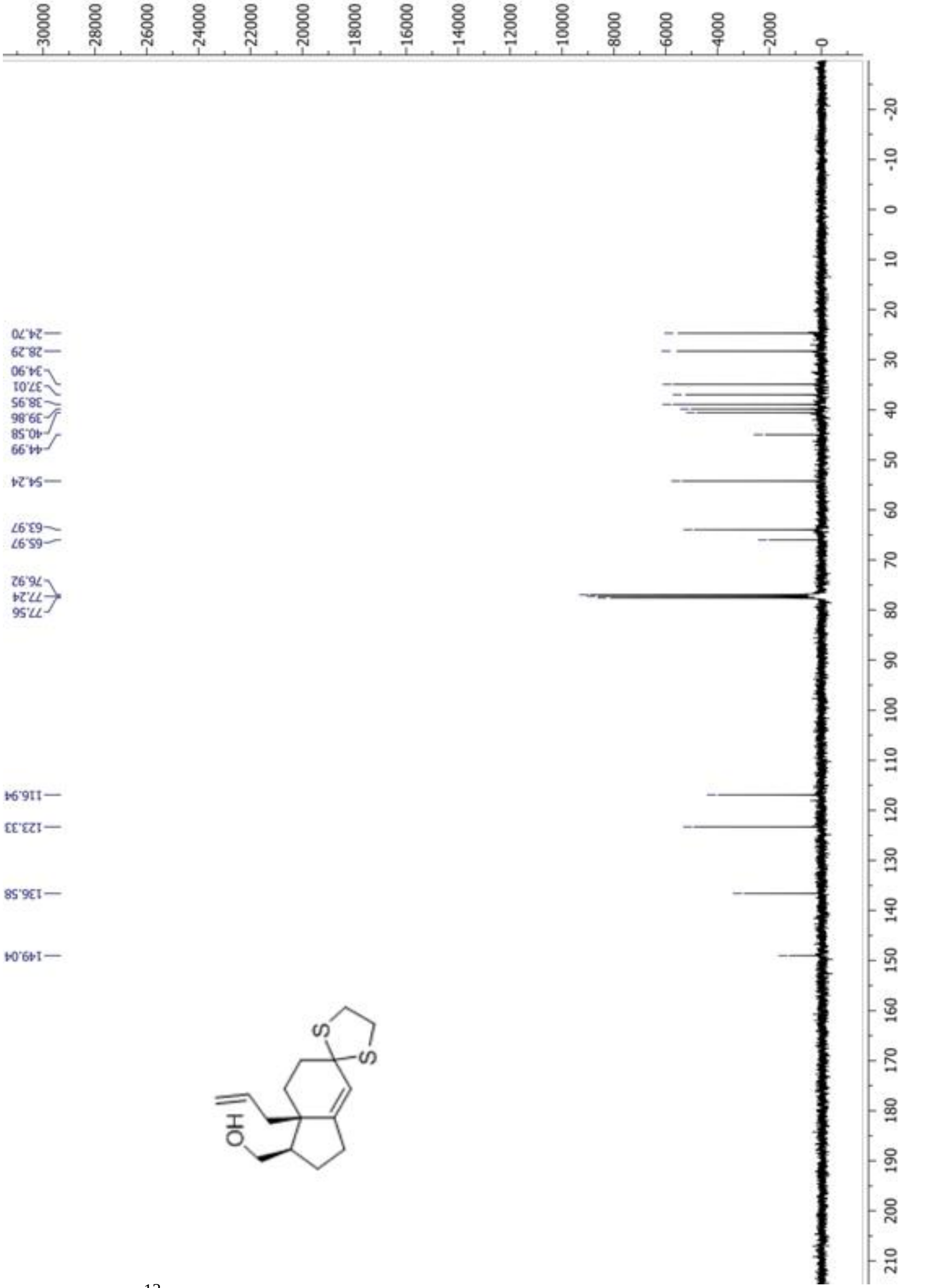

Spectrum 4. ^{13}C NMR ($CDCl_3$, 500 MHz) of compound **15**.

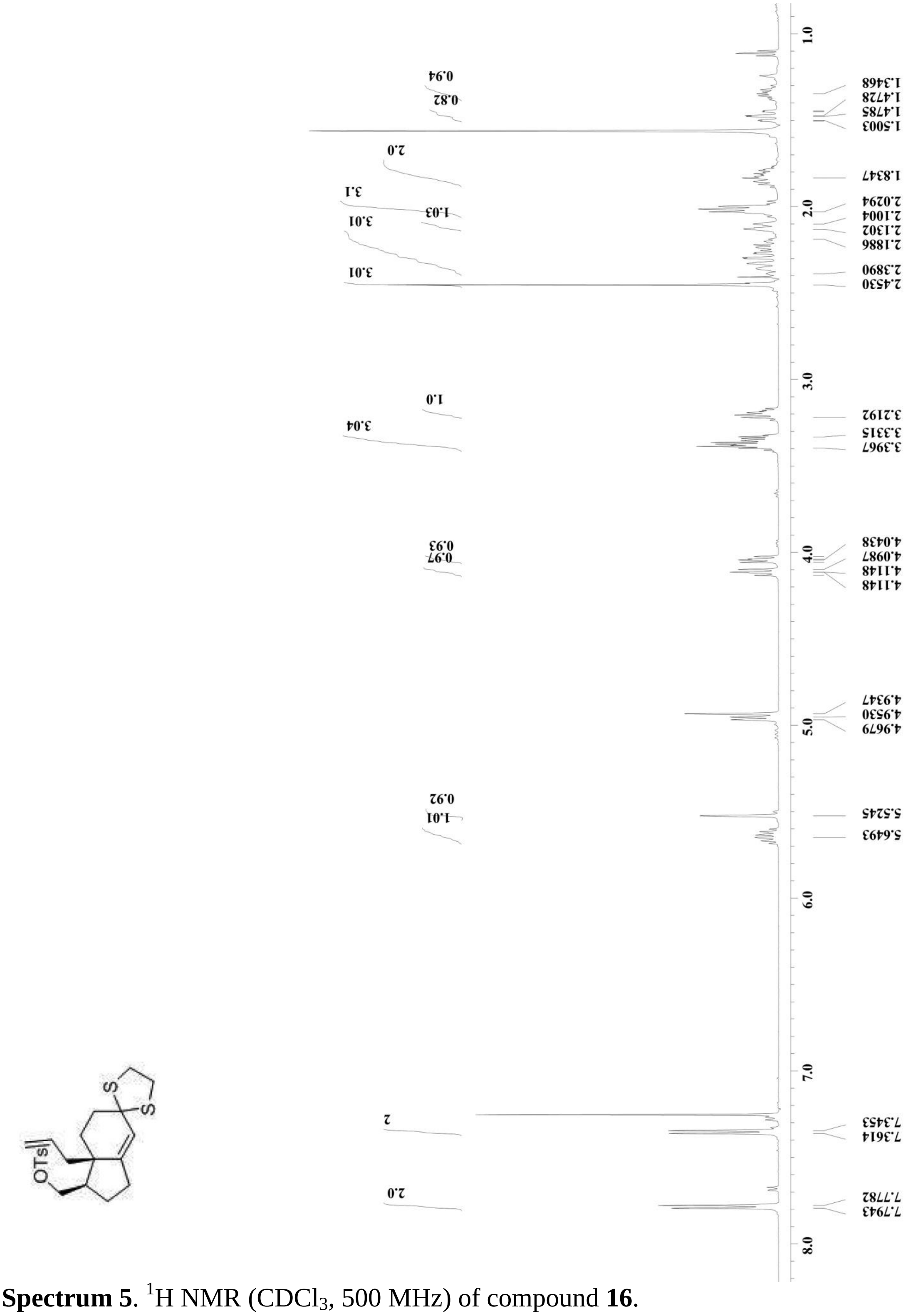

Spectrum 5. ^{1}H NMR ($CDCl_3$, 500 MHz) of compound **16**.

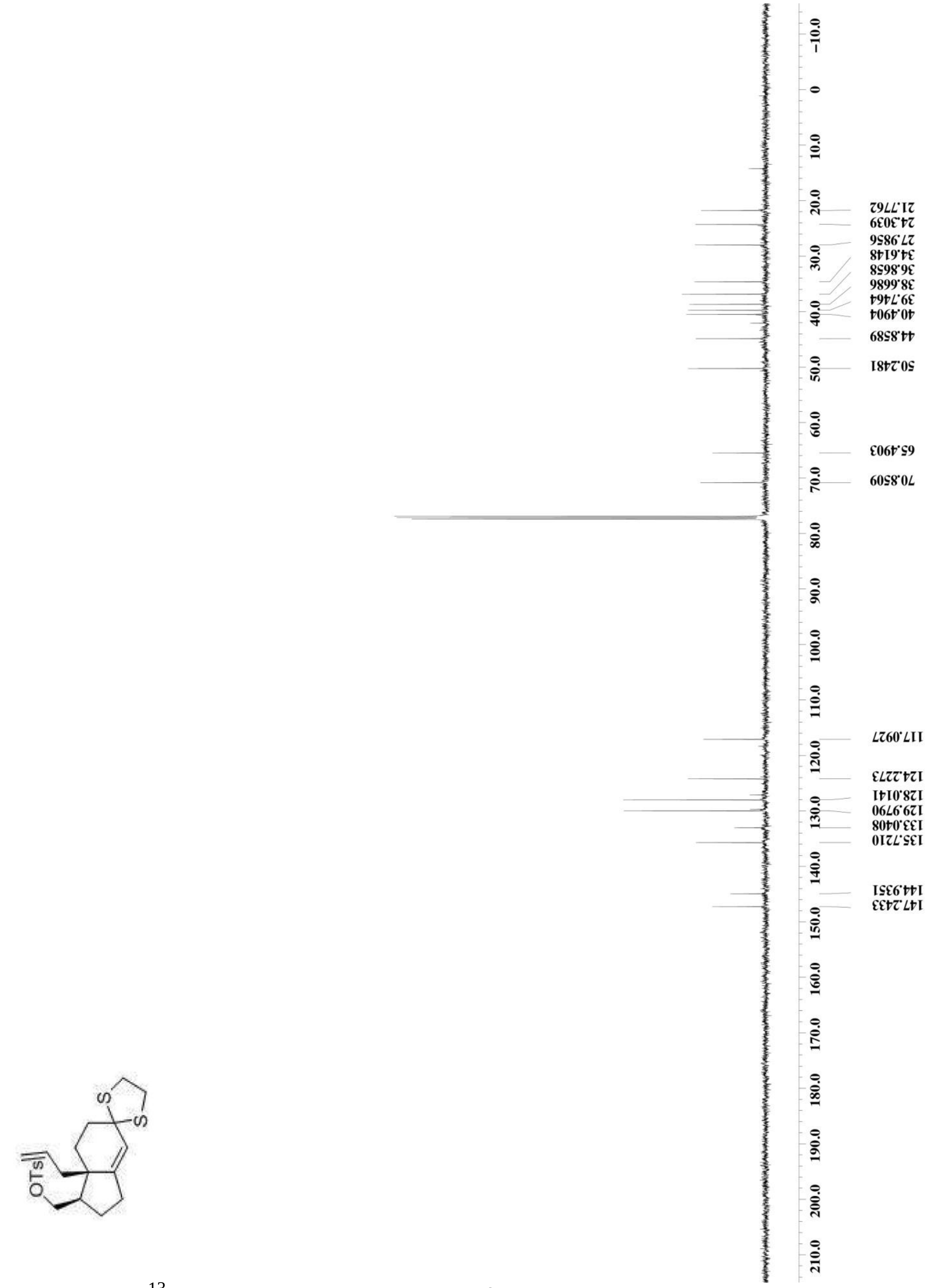

Spectrum 6. ^{13}C NMR ($CDCl_3$, 500 MHz) of compound **16**.

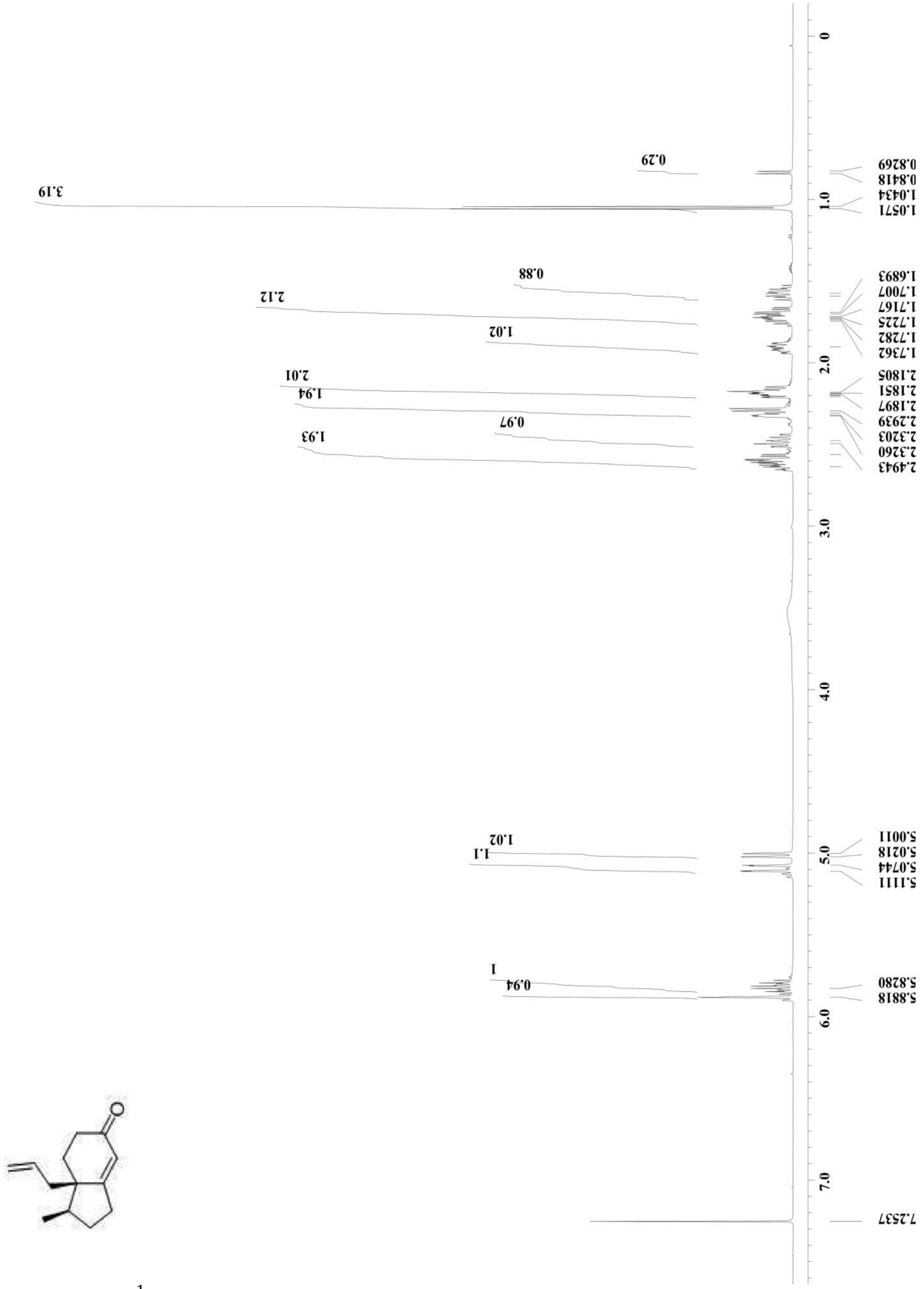

Spectrum 7. ^{1}H NMR ($CDCl_3$, 500 MHz) of compound **10**.

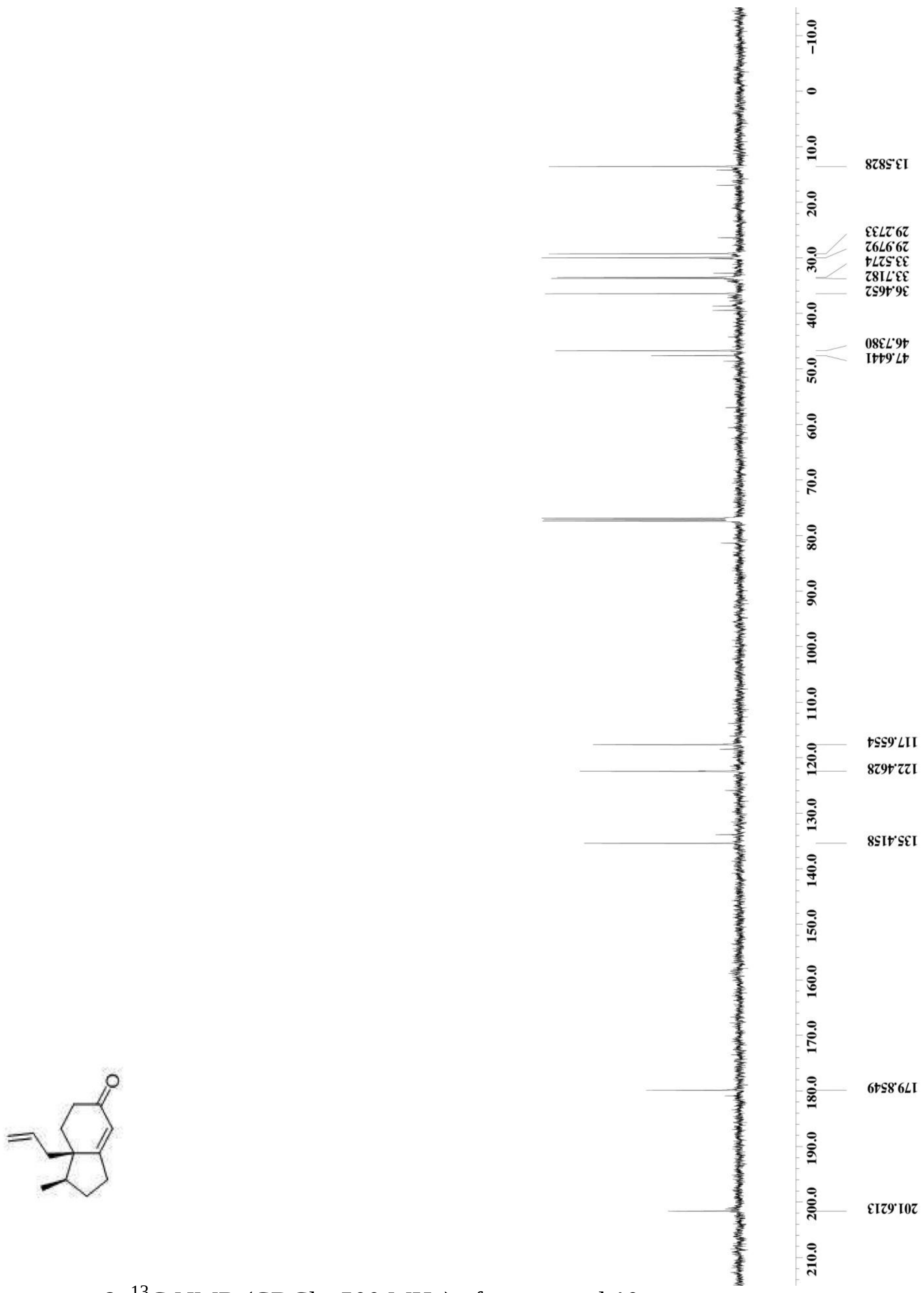

Spectrum 8. ^{13}C NMR ($CDCl_3$, 500 MHz) of compound **10**.

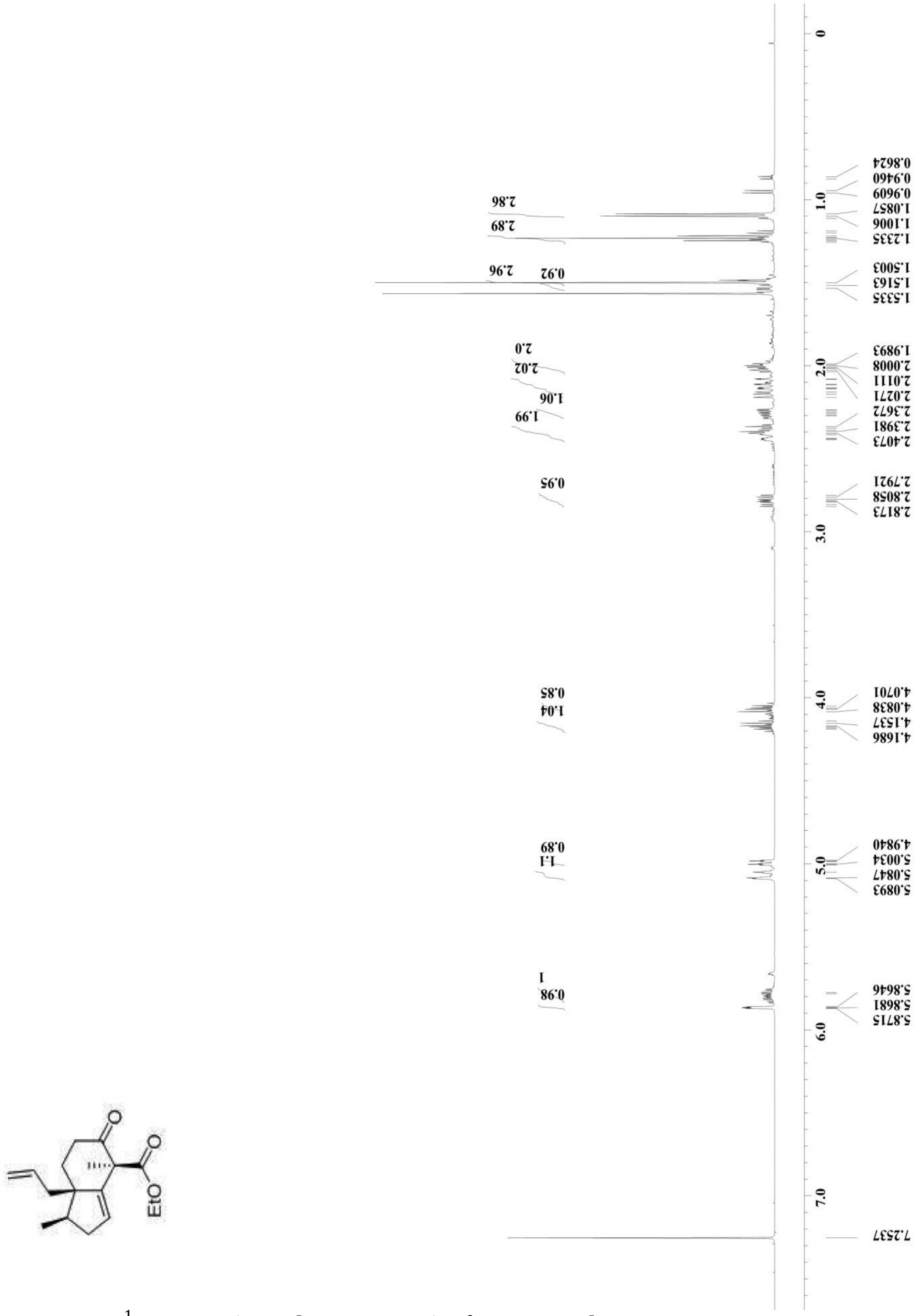

Spectrum 9. ^{1}H NMR ($CDCl_3$, 500 MHz) of compound **18**.

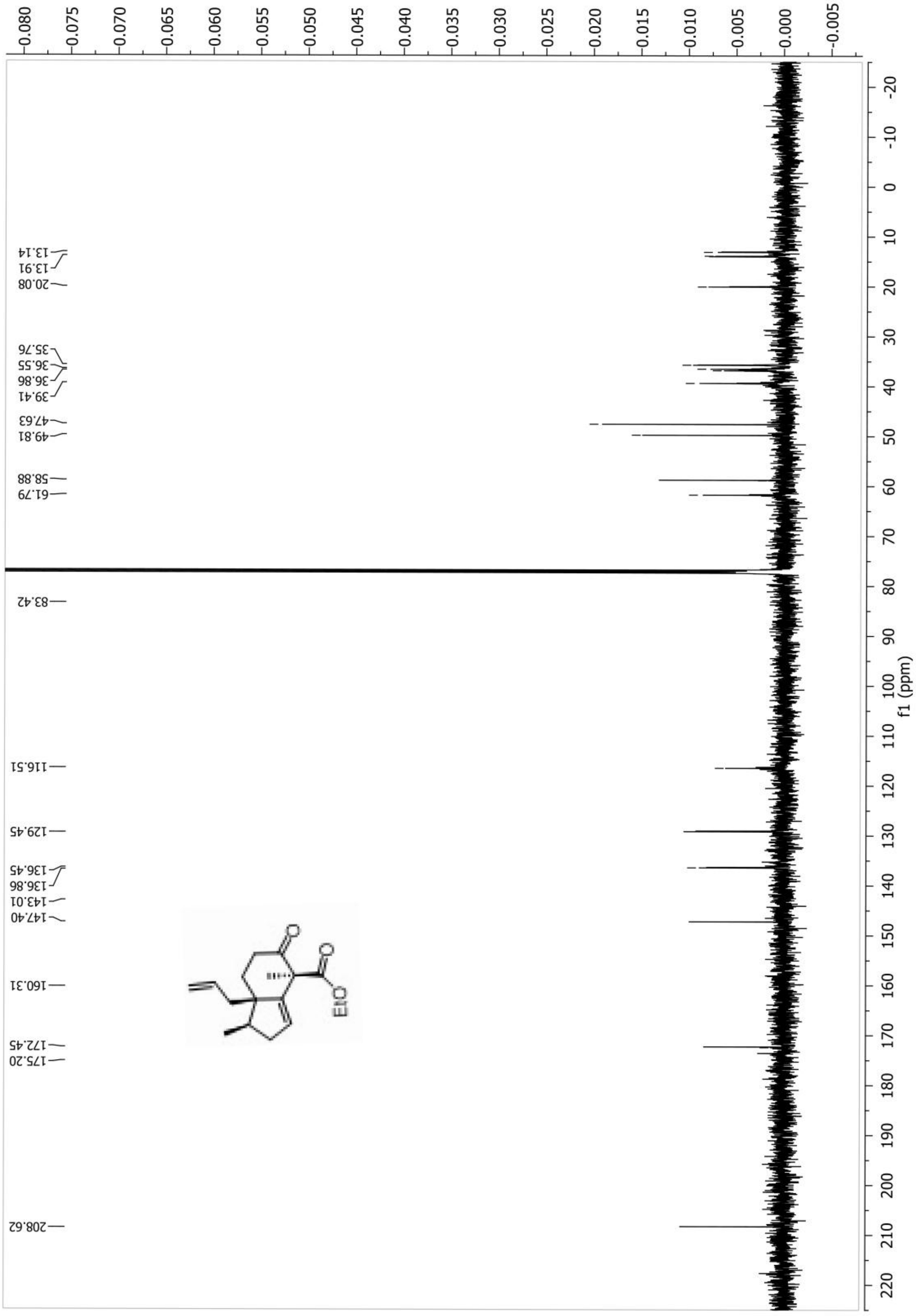

Spectrum 10. ^{13}C NMR ($CDCl_3$, 500 MHz) of compound **18**.

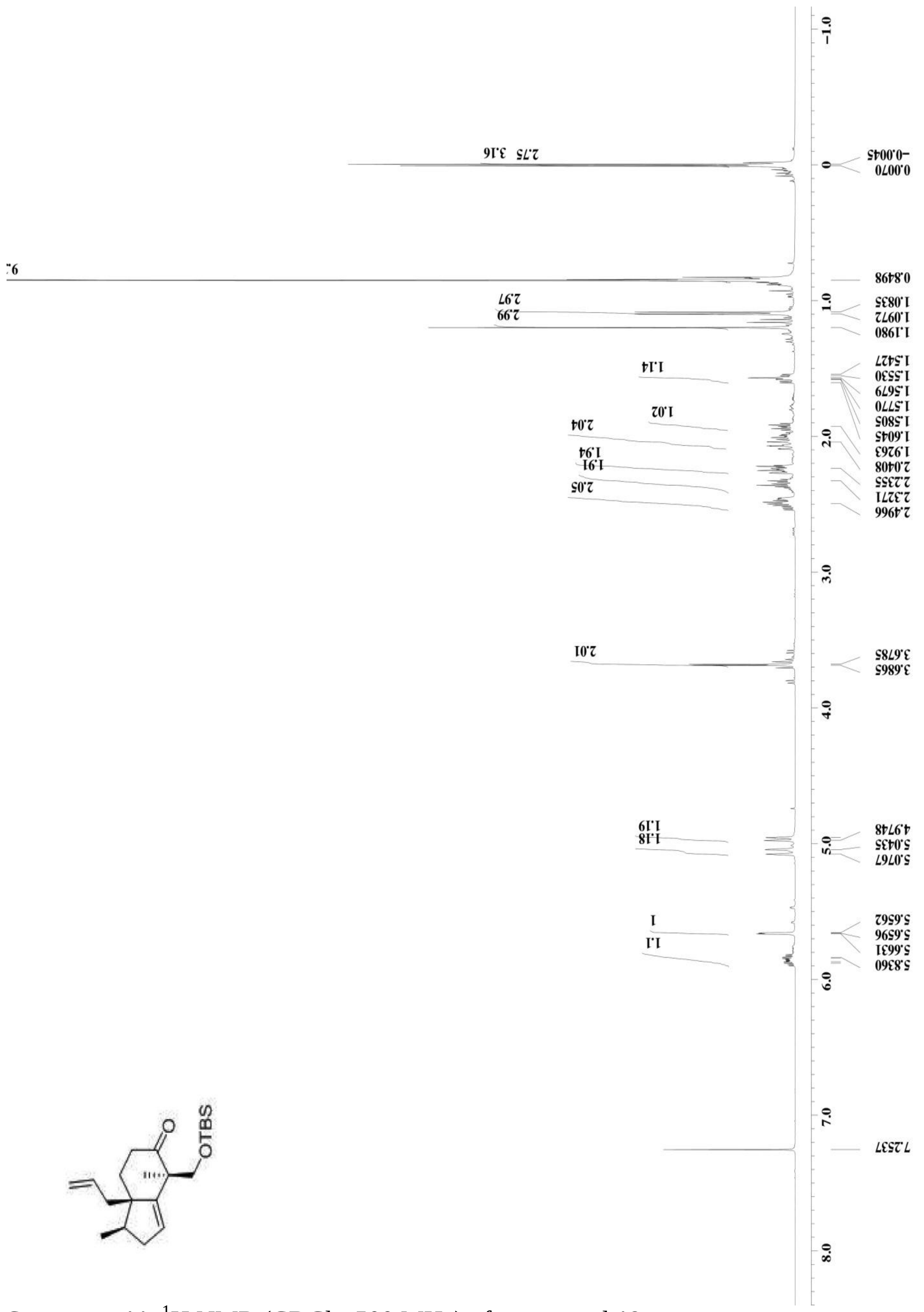

Spectrum 11. ^{1}H NMR ($CDCl_3$, 500 MHz) of compound **19**.

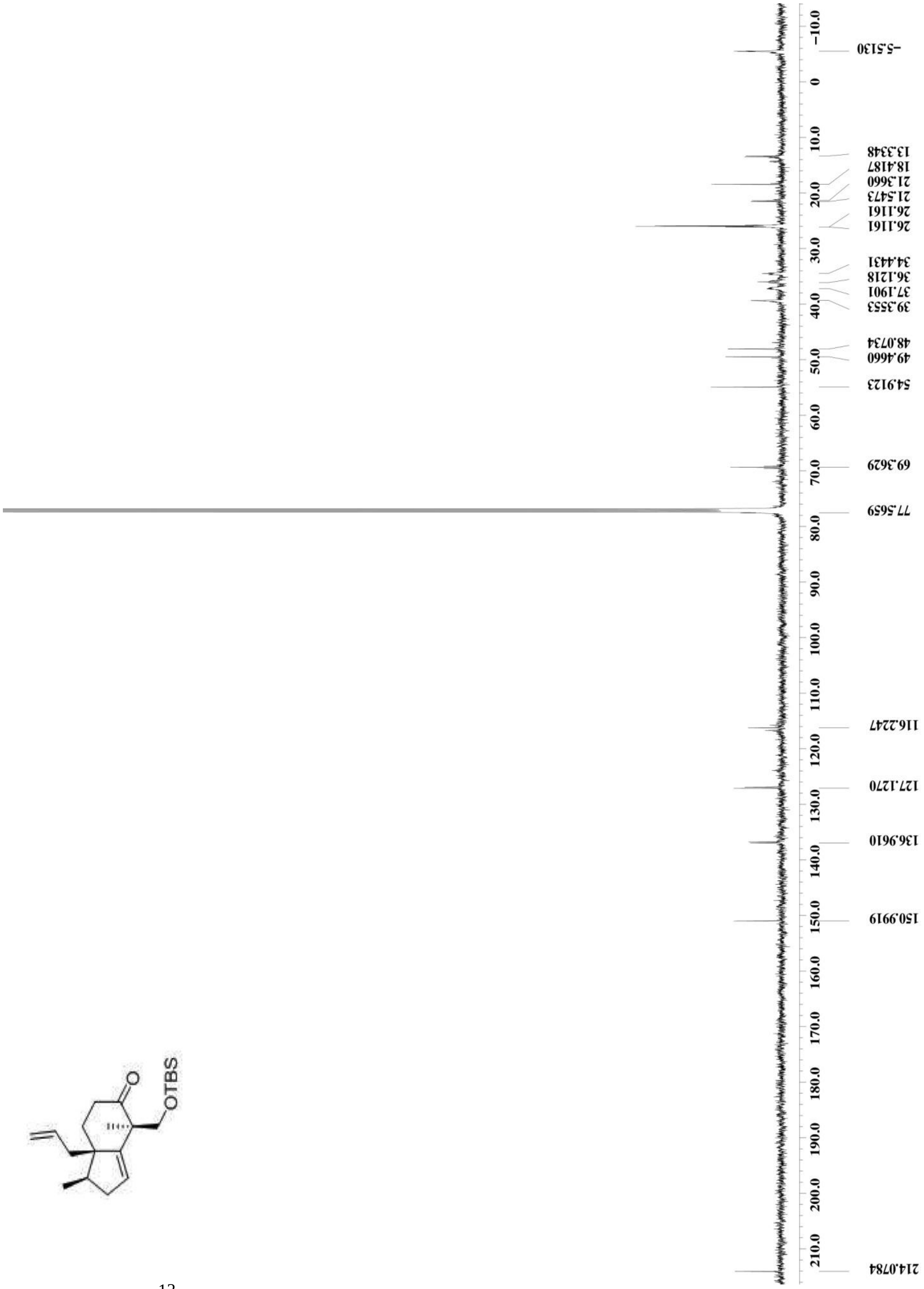

Spectrum 12. ^{13}C NMR ($CDCl_3$, 500 MHz) of compound **19**.

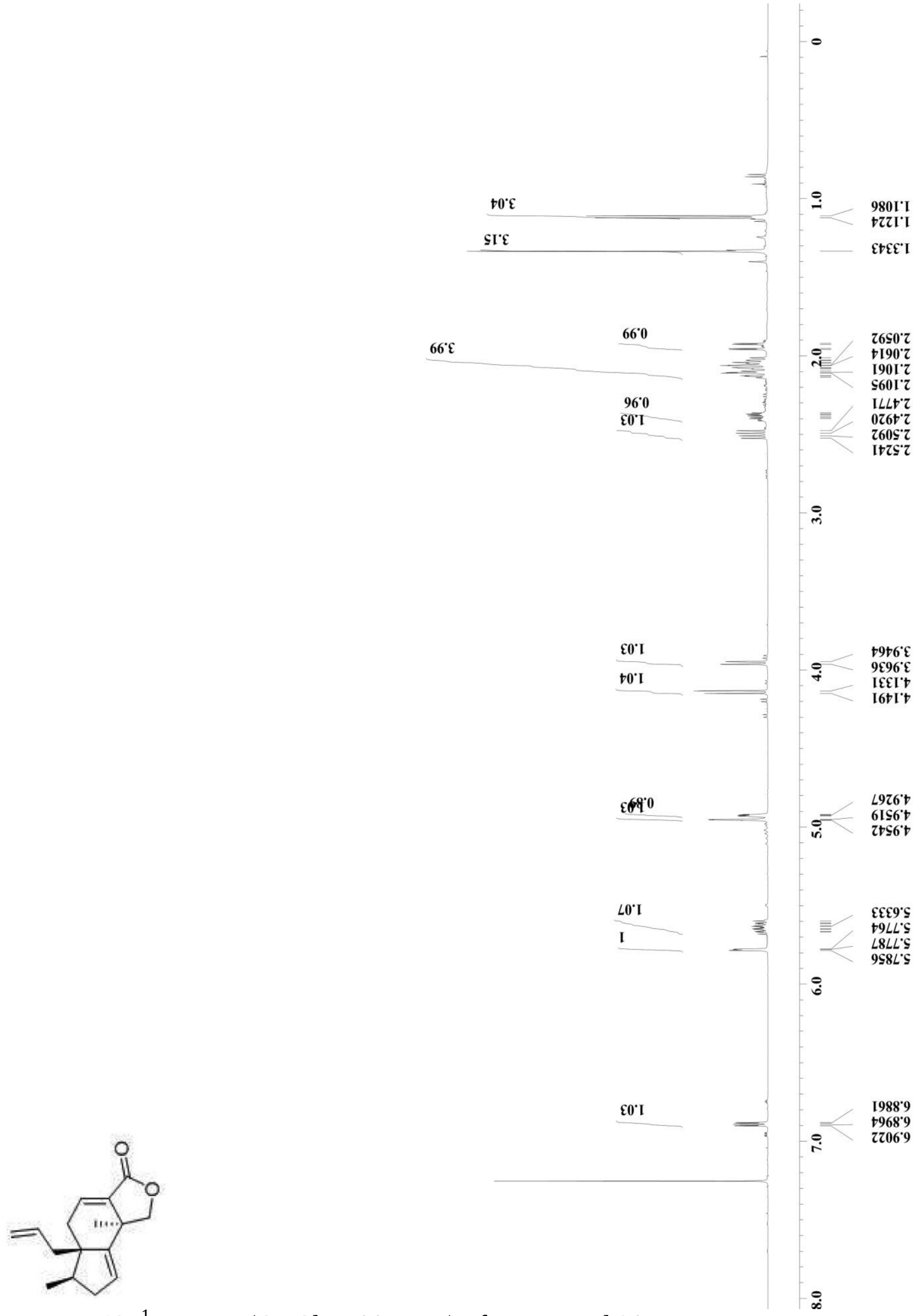

Spectrum 13. ^{1}H NMR ($CDCl_3$, 500 MHz) of compound **20**.

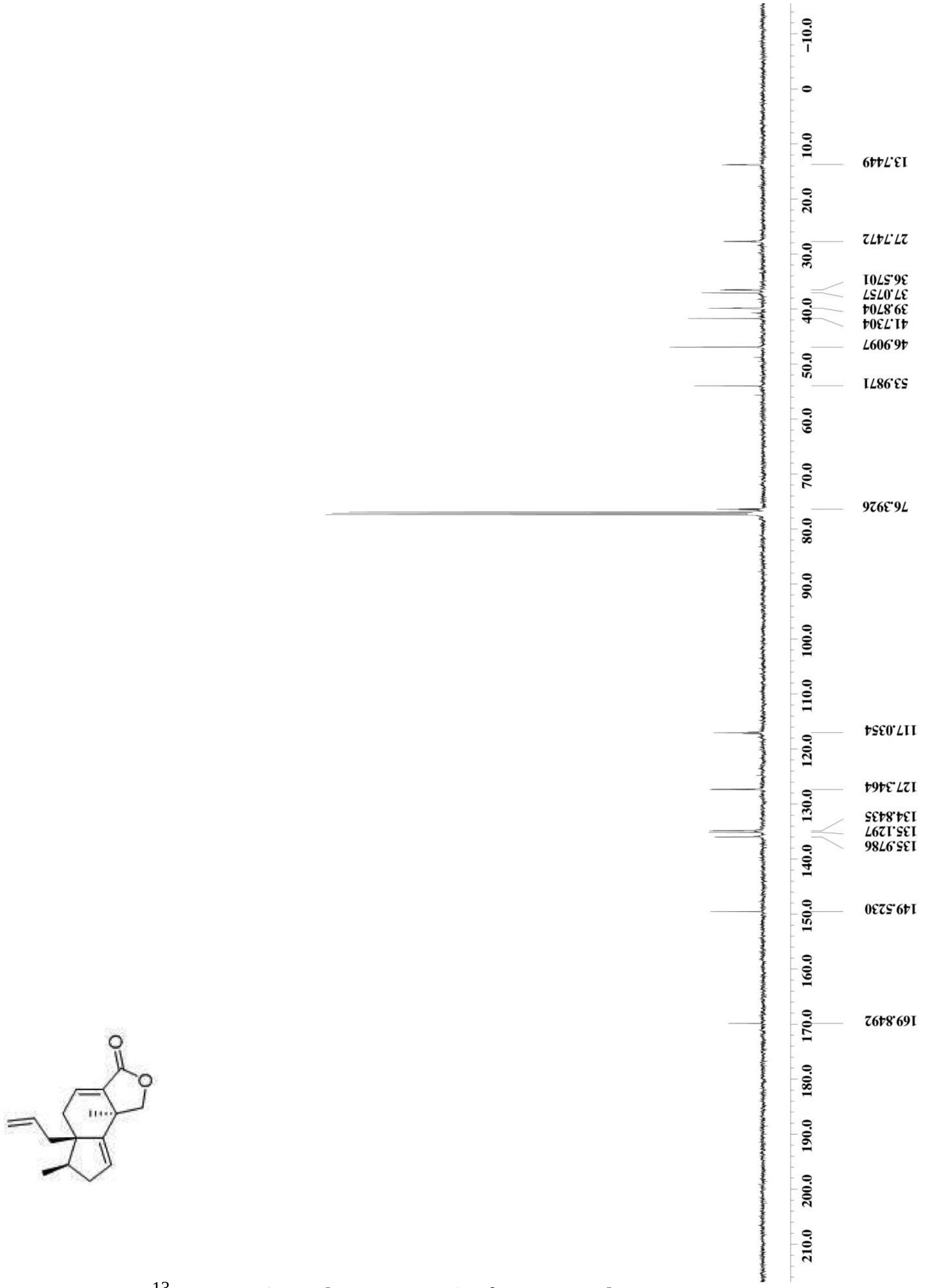

Spectrum 14. ^{13}C NMR ($CDCl_3$, 500 MHz) of compound **20**.

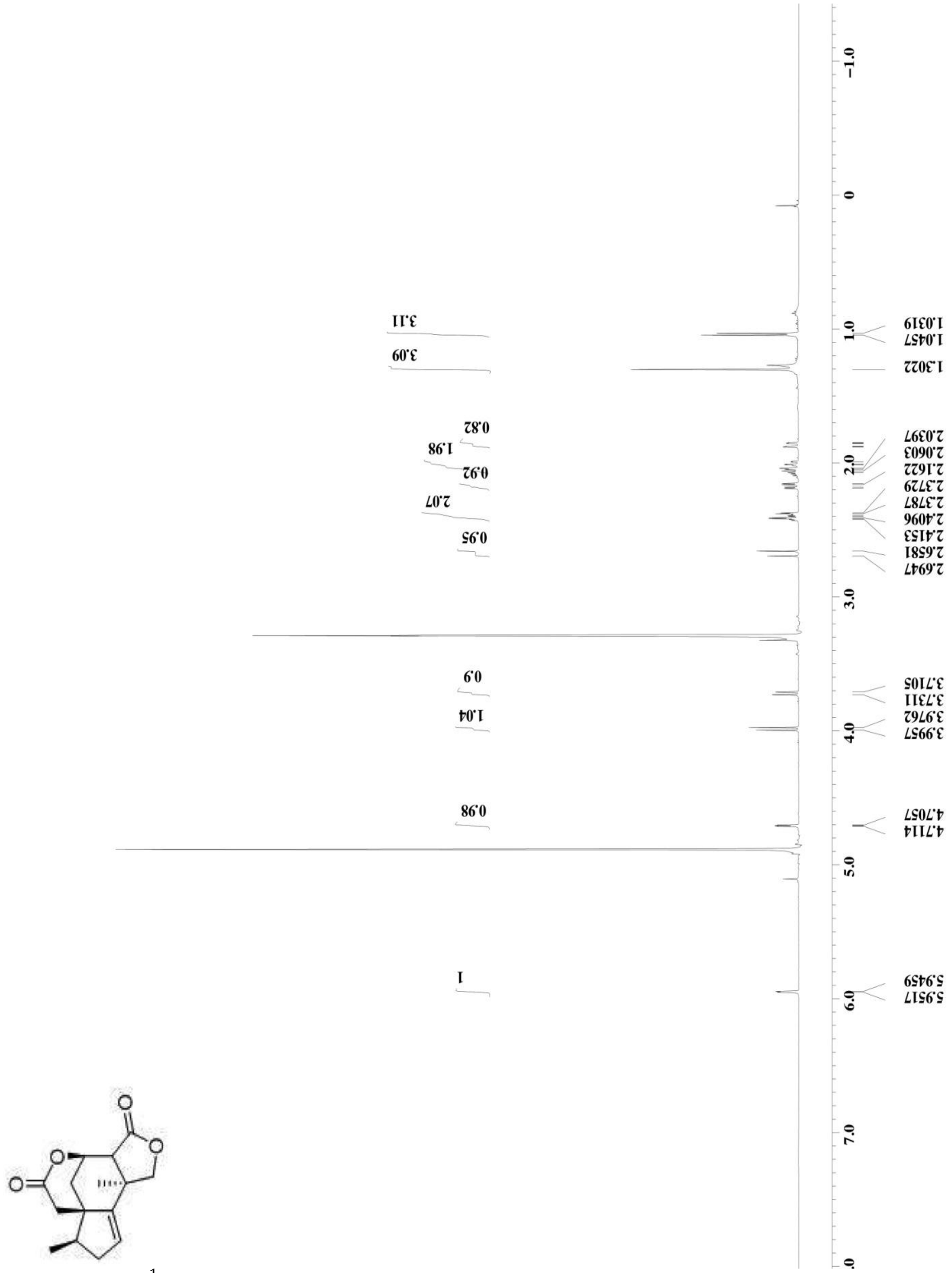

Spectrum 15. ^{1}H NMR (CD_3OD, 500 MHz) of compound **9**.

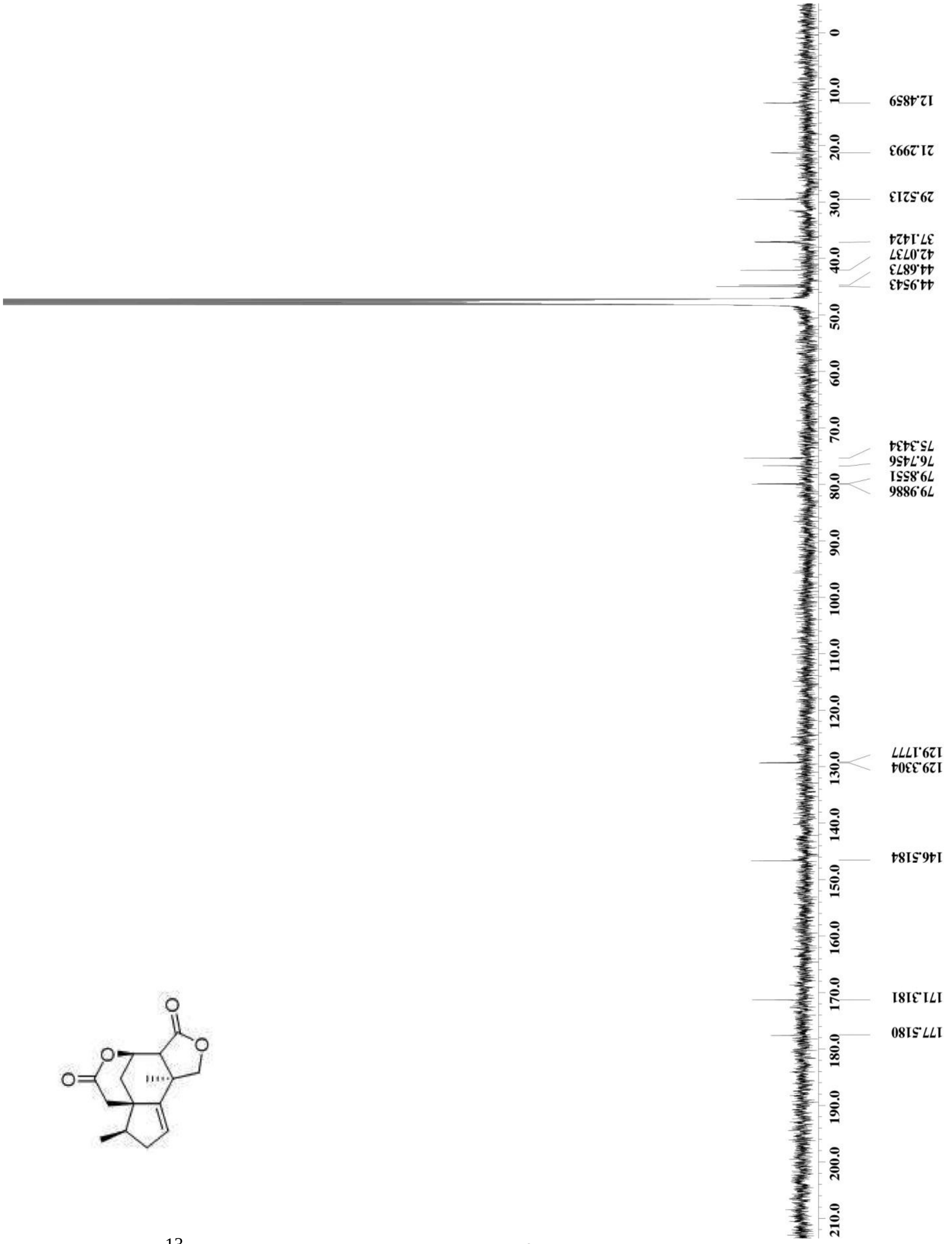

Spectrum 16. ^{13}C NMR (CD_3OD, 500 MHz) of compound **9**.
